# Borrelioses in Brazil: Is it time to consider tick-borne relapsing fever a neglected disease in Brazil?

**DOI:** 10.1590/0037-8682-0443-2021

**Published:** 2021-11-12

**Authors:** Álvaro A. Faccini-Martínez, Sebastián Muñoz-Leal, Marcelo B. Labruna, Rodrigo Nogueira Angerami

**Affiliations:** 1 University of Texas Medical Branch, Department of Pathology, Galveston TX, USA.; 2 Asociación Colombiana de Infectología, Committee of Tropical Medicine, Zoonoses and Travel Medicine, Bogotá, Colombia.; 3 Universidad de Concepción, Facultad de Ciencias Veterinarias, Departamento de Ciencia Animal, Chillán, Ñuble, Chile.; 4 Universidade de São Paulo, Faculdade de Medicina Veterinária e Zootecnia, Departamento de Medicina Veterinária Preventiva e Saúde Animal, São Paulo, SP, Brasil.; 5 Universidade Estadual de Campinas, Hospital de Clínicas, Seção de Epidemiologia Hospitalar, Campinas, SP, Brasil.

Dear Editor:

Since the early 1990s, some researchers in Brazil have argued in favor of an autochthonous Lyme-like borreliosis, locally known as Baggio-Yoshinari syndrome (BYS); however, the etiological agent of this disease has not been isolated or cultured from patients or clinical samples^1^. Conversely, a growing number of ecoepidemiological studies have reported the occurrence of tick-borne relapsing fever group borreliae (RFGB) in Brazil^2^ ^,^ ^3^. In this context, and from an epidemiological and medical perspective, two important questions arise: What is the actual scenario of borrelioses in Brazil and are *Borrelia* infections being neglected as human pathogens?

Tick-borne relapsing fever (TBRF) is a zoonotic infectious disease distributed worldwide and caused by pathogenic spirochaetes, included in the RFGB, transmitted to humans by ticks of the genus *Ornithodoros* ^4^. The ticks become infected while feeding on spirochaetemic vertebrates (e.g., mammals and birds) and maintain the infection for several weeks to years^4^. Humans are accidentally exposed to RFGB while entering environments where *Ornithodoros* spp. ticks occur, such as caves or dwellings with cracks in the walls^4^.

Clinically, after an incubation period of 4 to 18 days, patients typically present with an abrupt onset of fever between 38.7-40°C^4^. The first febrile episode, which is commonly accompanied by nonspecific symptoms such as headache, arthralgia, myalgia, and nausea, is usually the longest, lasting for an average of 3 days, and terminates with a crisis of shaking chills or rigors^4^. The initial febrile episode is followed by a series of relapses (1 to 13), corresponding to the peaks of spirochaetemia^4^. The average period between febrile episodes is seven days^4^. This is the typical relapsing fever pattern described during the course of non-fatal infections in the absence of antibiotic treatment^4^. 

In terms of public health, TBRF is considered a neglected bacterial infection, given the lack of distinguishing clinical features and the difficulty in disease diagnosing in regions where laboratory confirmation is lacking^5^. Consequently, TBRF is often attributed to more common etiologies such as malaria, typhoid, or dengue virus^5^. Nevertheless, studies in tropical regions of West Africa that investigated acute undifferentiated febrile illnesses (AUFI) have identified TBRF as an important etiology. For instance, between 2002 and 2004 in Togo, among febrile patients originally diagnosed and treated for malaria, the prevalence of TBRF was 8.8% (21/239), and 4.5% (7/154) of patients presented co-infection with malaria^6^. More recently, a study undertaken in Senegal has reported that TBRF cases accounted for 12% (94/800) of fever episodes in the Niakhar district between January and December 2016^7^. If we extrapolate this evidence to Latin America, could TBRF be an underlying etiology among the 21-68% (n=427) of AUFI reported in Brazil^8^?

**FIGURE 1:**
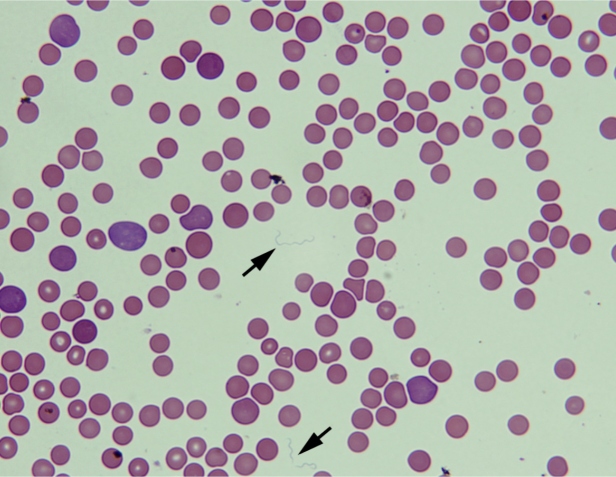
*Borrelia venezuelensis* in a peripheral blood smear (arrows), Giemsa staining at 1000× magnification (Source: Álvaro A. Faccini-Martínez, Sebastián Muñoz-Leal).

As a preliminary answer to the above-proposed question, recently, we reported five species of human-biting *Ornithodoros* ticks harboring RFGB in Brazil^2^ ^,^ ^3^. Briefly, in July 2017, 25 adult *Ornithodoros* specimens were collected from bird nest debris inside a hollow palm tree in the Riachão Municipality (Maranhão State)^2^. The ticks were identified as *Ornithodoros rudis* and fed on naïve vesper mice (*Calomys callosus*). Only one female tick was positive for borrelial infection, as determined by the observation of spirochetes in the blood of infected rodents on day 4 after tick exposure (Figure 1) and successful passage to a second host on day 3 of spirochaetemia. Phylogenetic analysis confirmed that the *Borrelia* sp. harbored by *O. rudis* from Maranhão State (*Borrelia venezuelensis* strain RMA01) represented a distinct lineage within the RFGB and was closely related to pathogenic *Borrelia turicatae* ^2^. In addition, between December 2018 and October 2019, new collections of *Ornithodoros* ticks were performed in natural ecosystems and inside human dwellings in six Brazilian states^3^. A total of 665 specimens belonging to eight species were collected and submitted for screening for *Borrelia* DNA. Four species of ticks were positive. An infection rate of 10.5% was noted in *Ornithodoros mimon* collected from a house in Cuiabá, Mato Grosso state, and a minimal infection rate (MIR) of 0.05% was detected in *Ornithodoros hasei* collected in a cave at Jericoacoara National Park, Ceará state. Besides, MIRs of 0.1% and 0.2% were detected in *Ornithodoros rietcorreai* and *Ornithodoros tabajara*, respectively, both collected between rocks at Serra das Almas Natural Reserve, Ceará state^3^.

On the other hand, pro-BYS researchers have promoted their in-house diagnostic criteria to support alleged Brazilian Lyme-like borreliosis^9^. They suggested the following criteria as "major parameters": 1) tick bite, contact with wild or domestic animals in risk areas, 2) erythema migrans or arthritis, neurological abnormalities, cardiac involvement, and 3) positive serology for *Borrelia burgdorferi* ^9^. Additionally, the following were suggested as "minor parameters": 1) recurrent episodes, 2) chronic fatigue, myalgia, arthralgia, cognitive disorder, paresthesia of extremities, and 3) identification of motile spirochete-like structures by dark-field microscopy or Giemsa staining^9^. Thus, a BYS case is defined in the presence of three major parameters, or two major and two minor parameters^9^. 

Interestingly, most diagnostic criteria for BYS significantly overlap with TBRF. For example, pathogenic RFGB are transmitted by the bite of *Ornithodoros* ticks^4^, and some of these spirochetes exhibit neurotropism, thus causing neurological abnormalities^4^. Moreover, it is known that patients infected with RFGB develop recurrent febrile episodes^4^, and can yield a positive result in serological tests developed to diagnose Lyme borreliosis^10^. To support a TBRF diagnosis, motile spirochetes must be identified by dark-field microscopy or Giemsa staining of a peripheral blood smear^4^. In addition, and importantly, among patients from Lyme borreliosis non-endemic areas or areas with low disease incidence (such as Brazil)^1^, one must rule out other etiologies that could cause cross-reactions in *B. burgdorferi* serological tests (e.g., infectious, hematologic, or rheumatological conditions) and nonspecific symptoms (e.g., chronic fatigue, myalgia, arthralgia, cognitive disorder, paresthesia of extremities)^11^.

In order to raise awareness of TBRF among health professionals, it is crucial to consider the four factors proposed by Dworkin and co-workers that could contribute to poor detection of this infectious disease^12^: 1) the inexperience of the microscopist (spirochetes misidentified as artifacts), 2) lack of suspicion for relapsing fever, 3) increased use of automated blood count rather than manual blood smear, and 4) examination of blood during the asymptomatic interval when spirochetes are absent from the circulation or are below the level of detection. 

Finally, to better understand the current scenario regarding borrelioses in Brazil, increased clinical suspicion and improved training for laboratory diagnosis are necessary. The above must be supported by a surveillance system that includes case definition criteria based on clinical, laboratory, and ecoepidemiological features that are scientifically validated.
